# INCREASED TEMPORAL GAIT VARIABILITY OVER TIME IS RELATED TO FUTURE DEMENTIA

**DOI:** 10.1093/geroni/igad104.0549

**Published:** 2023-12-21

**Authors:** Qu Tian, Susan Resnick, Eleanor Simonsick, Luigi Ferrucci

**Affiliations:** National Institute on Aging, National Institutes of Health, Baltimore, Maryland, United States; National Institute on Aging, National Institutes of Health, Baltimore, Maryland, United States; National Institute on Aging, National Institutes of Health, Baltimore, Maryland, United States; National Institute on Aging, National Institutes of Health, Baltimore, Maryland, United States

## Abstract

Age-related brain changes affect the timing and coordination of gait. High gait variability may stem from impaired brain motor control and has been associated with compromised cognition and cognitive impairment. Whether increased gait variability over time is associated with future MCI or dementia is unknown. In the Baltimore Longitudinal Study of Aging, we used age-, sex-, and race-adjusted linear mixed-effects models to examine whether changes in gait variability were associated with future MCI or dementia in participants aged 65+. Gait characteristics were quantified by 3D motion analysis. Independent of baseline gait speed and compared to cognitively normal participants (n=387), those who developed dementia (n=24) had greater rates of increase in gait cycle time variability, stance time variability, and swing time variability (but not mean performance) before the symptom onset (all p<0.05). Further adjustment for concurrent assessment of visuospatial ability or manual dexterity attenuated these associations. Adjustments for verbal memory, psychomotor speed, attention, or executive function did not attenuate these associations. Rates of changes in these gait variability measures were not statistically different between cognitively normal participants (n=387) and those who developed MCI (n=41). Baseline gait variability measures were not different across groups. These findings suggest that longitudinal trajectories of gait variability, not one-time assessment, provide the additional predictive value of future dementia over and beyond gait speed. Mechanisms may include compromised visuospatial ability or manual dexterity. Future neuroimaging studies are warranted to understand neural substrates connecting increased gait variability and dementia risk.

